# Ultrasound- and Colour Doppler-Guided WALANT Surgery for Insertional Achilles Tendinopathy: A Prospective Case Series on 53 Consecutive Patients

**DOI:** 10.3390/jfmk11010034

**Published:** 2026-01-15

**Authors:** Philip Bazala, Markus Waldén, David Roberts, Christoph Spang, Håkan Alfredson

**Affiliations:** 1 Capio Ortho Center Skåne, 21532 Malmö, Swedenmarkus.walden@telia.com (M.W.);; 2 Unit of Public Health, Department of Health, Medicine and Caring Sciences, Linköping University, 58183 Linköping, Sweden; 3 Private Orthopaedic Spine Center, 97080 Würzburg, Germany; christoph.spang@dr-alfen.de; 4 Institute of Sports Science, Würzburg University, 97082 Würzburg, Germany; 5 Sports Medicine Unit, Department of Community Medicine and Rehabilitation, Umeå University, 90187 Umeå, Sweden; 6 Alfredson Tendon Clinic, Capio Ortho Center Skåne, 21532 Malmö, Sweden

**Keywords:** athletic injuries, pain, sports, tendon

## Abstract

**Background:** Treatment of chronic painful insertional Achilles tendinopathy is known to be challenging. If non-surgical treatment does not give sufficient relief of symptoms, surgery may be indicated. Treatment with ultrasound (US)- and colour Doppler (CD)-guided wide-awake-local-anaesthetic-no-tourniquet (WALANT) surgery for insertional Achilles tendinopathy is a new approach with promising clinical results. This study aimed to evaluate clinical results of this new approach on patients suffering from insertional Achilles tendinopathy. **Methods:** Forty-eight consecutive patients with 53 symptomatic tendons (33 men with 34 tendons, mean age 49.3 ± 12.0 years; 14 women with 18 tendons, mean age 55.0 ± 7.4 years) and a duration of more than 12 months with painful insertional Achilles tendinopathy (including tendon, bursae, bone, and plantaris pathology) were included. US- and CD-guided WALANT surgery with removal of pathological bursae, bone, and tendons was used. Immediate weight-bearing loading was allowed, followed by a structured rehabilitation protocol for the first 12 weeks after surgery. VISA-A scores before and after surgery and a questionnaire that evaluated subjective satisfaction with the treatment and current activity level were used. **Results:** In total, 42/48 patients with 46/53 tendons participated in a 3-year follow-up (mean 34 ± 9 months) by an independent examiner; 39/42 patients with 43/46 tendons were satisfied (*n* = 37) with the treatment. The mean VISA-A score increased significantly from 41.9 ± 18.2 pre-operatively to 87.7 ± 18.2 post-operatively (*p* < 0.001). There were three surgical complications, two superficial wound infections, and one minor wound rupture. **Conclusions:** Patients who suffered from chronic painful insertional Achilles tendinopathy treated with US- and CD-guided WALANT surgery followed by immediate weight-bearing showed high patient subjective satisfaction rates and better functional scores at the 3-year follow-up with a low complication rate. This novel treatment approach warrants more study, including randomised trials comparing it against traditional surgical procedures according to Nunley and Keck and Kelly.

## 1. Introduction

Insertional Achilles tendinopathy is seen among elite and recreational athletes and sedentary individuals [1,2,3]. The condition is considered difficult to treat [2,4,5,6], not only because it includes pathology in the distal Achilles tendon itself, but also in the superficial and deep bursae and bone tissue (Haglund deformity, intra-tendinous bone spurs, and prominences, etc.) [7,8]. Radiographs and magnetic resonance imaging (MRI) are frequently used diagnostic tools, but ultrasound (US) is the preferred examination device for tendons and tendon insertions [9].

Innervation patterns have not been fully clarified, but a recent study showed the subcutaneous bursa to have the most sensory nerves in the region compared to, for example, the Achilles tendon, the retro-calcaneal bursa, and bone in patients with chronic painful insertional Achilles tendinopathy [7]. Furthermore, the plantaris tendon has recently been highlighted as an involved structure in insertional Achilles tendinopathy [10,11].

Conservative treatment options are eccentric exercise and combined treatments including extracorporeal shockwave therapy [12]. In many cases, surgery is needed. Surgical interventions in use for insertional Achilles tendinopathy include several different open surgical procedures under spinal or general anaesthesia ranging from removal of the retro-calcaneal bursa and resection of the upper calcaneus [13] to more invasive methods like tendon detachment, such as according to Nunley, and osteotomy procedures, such as according to Keck and Kelly [14,15,16,17,18]. A more novel approach is US- and colour Doppler (CD)-guided wide-awake local anaesthetic no tourniquet (WALANT) surgery with immediate weight-bearing and without immobilisation for insertional Achilles tendinopathy, which has shown promising clinical results in two recent studies [19,20].

This study aimed to evaluate the clinical results after this new approach with US- and CD-guided WALANT surgery on patients suffering from insertional Achilles tendinopathy.

## 2. Materials and Methods

### 2.1. Patients

In total, 48 consecutive patients with 53 symptomatic tendons (29 left, 24 right), including 33 men with 34 tendons (mean age 49.3 ± 12.0 years) and 14 women with 18 tendons (mean age 55.0 ± 7.4 years) underwent operations between September 2020 and February 2023 at the Alfredson Tendon Clinic, Capio Ortho Center Skåne, Malmö, Sweden. All patients were >18 years old and were fluent in Swedish. The inclusion criterion was a long duration with insertional Achilles pain without effective conservative treatment. All had insertional Achilles tendon pain for at least 12 months and were non-responsive to rest and non-surgical treatment (most had tried eccentrics). They suffered from insertional pain during Achilles tendon loading activity and had palpable tenderness at the back of the heel. Exclusion criteria were (1) previous surgery of the Achilles insertion, (2) previous fractures of the calcaneus or ankle joint, and (3) systemic chronic inflammatory conditions affecting the foot and/or ankle joint.

The activity level of these patients ranged from high level/elite sports—running (*n* = 11), decathlon (*n* = 1), triathlon (1), football (*n* = 1), ice hockey (*n* = 1)—to recreational activities—jogging (*n* = 11), padel (*n* = 4), tennis (*n* = 2), biking (*n* = 2), and walking (*n* = 19). These patients were generally healthy, but seven patients suffered from hypertension, two had type 2 diabetes and one had hyperlipidaemia. There was only one smoker. All patients had tried treatment with non-steroid anti-inflammatory drugs (NSAIDs), and six patients had been treated by local cortisone injection. 

### 2.2. Pre-Operative Examination

All examinations were carried out by an experienced orthopaedic surgeon working almost exclusively with various tendon-related conditions.

Clinical examination: There was a tender thickening at the back of the heel and a widened distal part of the Achilles tendon in all patients. There was also a suspected filling in the region of the retro-calcaneal bursa, local tenderness at any bony prominences, and sometimes a local tenderness in the region of the plantaris tendon on the medial aspect of the insertion.

**Figure 1:**
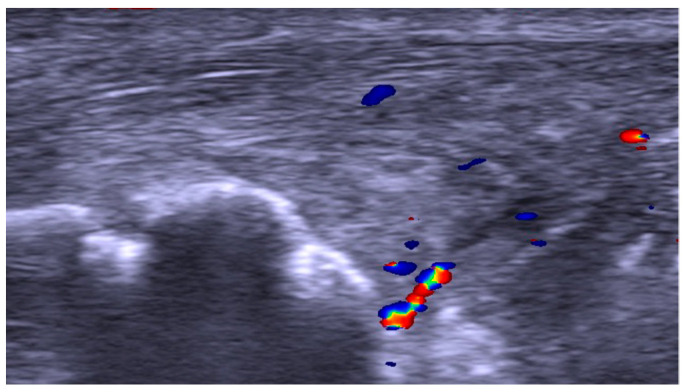
Ultrasound and colour Doppler image showing enlarged retro-calcaneal bursa with localised high blood flow (colour), thickened tendinopathic distal Achilles tendon, and Haglund-like deformity of the upper calcaneus.

**Figure 2:**
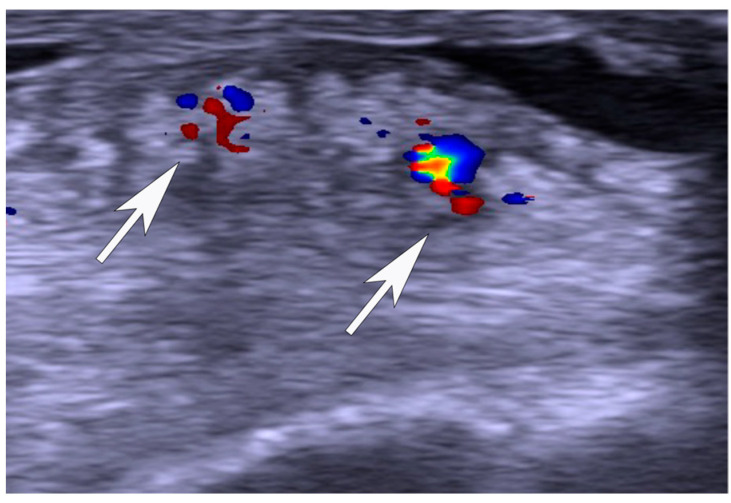
Ultrasound and Doppler image that shows enlarged and thickened subcutaneous bursa with localised high blood flow in deep side of the bursa (arrows).

**Figure 3:**
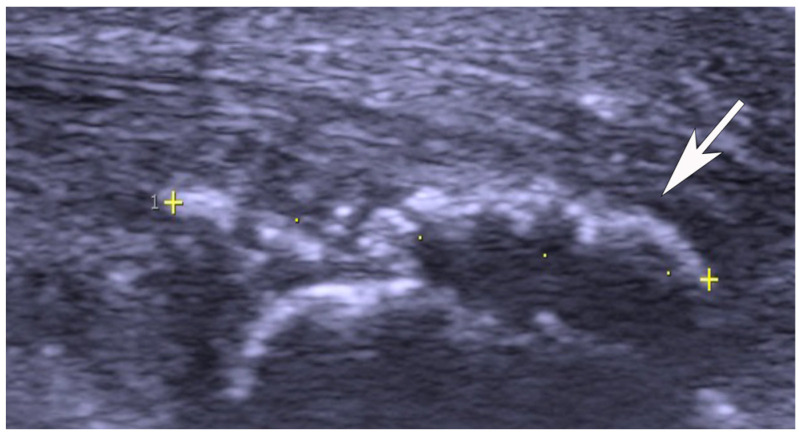
Ultrasound and colour Doppler image showing calcifications and bone formations inside the tendinopathy of the Achilles insertion (arrow and cross).

US and CD examinations: High-resolution grey scale US and CD examinations (SECMA AB, Askim, Sweden) with a linear multi-frequency (8–13 MHz) probe were used in order to verify the diagnosis indicating pathology in the Achilles insertion (Figure 1, Figure 2 and Figure 3). The most typical findings were combined pathology including enlarged subcutaneous and retro-calcaneal bursae with high blood flow within the bursa walls; a thickened distal part of the Achilles tendon, including structural tendon changes located anteriorly and centrally in the tendon with localised high blood flow inside and outside the anterior part of the tendon; a prominent upper calcaneus (true Haglund or Haglund-like deformity); and a thickened plantaris tendon insertion surrounded by high blood flow in 25 tendons. There was combined pathology including enlarged subcutaneous bursa with high blood flow within the bursa and a thickened plantaris tendon insertion surrounded by high blood flow in twelve tendons. Combined pathology included enlarged subcutaneous bursa with high blood flow inside the bursa and bone spurs and/or bone prominences inside the distal tendon in seven tendons. Isolated pathology in an enlarged subcutaneous bursa and high blood flow inside the bursa alone was identified in nine tendons.

### 2.3. Surgical Procedure

The technical procedure has been described in detail in previous reports [19,20]. Briefly, wide-awake local anaesthetic no tourniquet (WALANT) surgery was used for all operations. After the disinfection of the skin with wet cloths of chlorhexidine cutaneous solution (Klorhexidionsprit 5 mg/mL, Fresenius Kabi, Bad Homburg, Germany), 5–10 mL of a local anesthetic (Xylocain+ adrenalin 10 mg/mL + 5 μg/mL, Aspen, uMhlanga, South Africa) was infiltrated subcutaneously, inside and around the subcutaneous and retro-calcaneal bursae, towards the periosteum of the upper calcaneus, and anteriorly of the distal Achilles tendon. The skin was then scrubbed and draped using a sterile paper cover exposing only the heel and the distal portion of the Achilles tendon. The surgical procedure started 10–15 min after local anaesthesia was injected. Via a lateral and/or medial (medial if the plantaris tendon looked pathological) 4–5 cm longitudinal incision into the skin, subcutaneous tissues were visualized. The following steps were carried out depending on the pathology. First, for the subcutaneous bursa, the posterior part of the bursa was carefully dissected from the skin with a scalpel and scissors and then the anterior part of the bursa was separated from the tendon before the whole bursa was finally removed. Second, for the retro-calcaneal bursa, this bursa was made visible by lifting the Achilles tendon posteriorly with a retractor, and then the bursa was carefully dissected with scalpel and scissors from the anterior side of the tendon and removed. Third, for the prominent upper calcaneus, again by lifting the Achilles tendon posteriorly, the upper calcaneus was identified and removed by a small osteotome. Any remaining bony impingement could be ruled out by placing the index finger between the tendon and upper calcaneus and dorsiflexing the ankle joint. Fourth, for the Achilles tendinopathy, the infiltrating fat tissue on the anterior side of the distal Achilles tendon was scraped away using a scalpel. Fifth, if there was a plantaris tendon involvement suspected, an incision on the medial aspect of the insertion was performed, and the distal part (4–5 cm) of the plantaris was carefully released with scissors and removed. Sixth, for intra-tendinous bone spurs and bone prominences, a longitudinal tenotomy was used to remove the local bone using the small osteotome. The tenotomy was sutured side to side using 4/0 Vicryl sutures.

Finally, the area was flushed with 3–4 mL of Marcain (5 mg/mL, Aspen, South Africa). Following careful hemostasis using bi-polar diathermia, the skin was then closed by single non-resorbable sutures. They were removed after 3 weeks. Soft dressing and elastic wrapping for the lower leg and foot were applied.

### 2.4. Post-Operative Rehabilitation

A strict and structured rehabilitation protocol that lasted for 12 weeks was followed before allowing jogging/running and sport-specific exercises, as outlined in Table 1.

### 2.5. Ethics

This study was conducted in accordance with the Declaration of Helsinki and approved by the Swedish Ethical Review Authority (reference number 2022-02889-01). Written informed consent was collected from all patients included in this follow-up study.

### 2.6. Follow-Up

An independent examiner (a foot and ankle surgeon) without any previous contact with study patients and who did not participate in initial patient examinations, surgeries, or rehabilitation, was responsible for the follow-up evaluation (P.B.). The self-administered Victorian Institute of Sports Assessment Achilles (VISA-A) score was filled out by patients on the day of surgery and at the follow-up. A study-specific questionnaire that evaluated subjective satisfaction with the surgical treatment (binary, satisfied or not satisfied) and the level of physical activity was completed only at the follow-up.

### 2.7. Statistical Analysis

Statistical analyses were performed using SPSS (version 30, Statistical Package of Social Science, SPSS Inc., Chicago, IL, USA). A paired Student’s *t*-test was used to analyse any differences in VISA-A scores before and after surgery. The level of significance was set to *p* < 0.05.

## 3. Results

In total, 42/48 patients with 46/53 tendons completed the follow-up at a mean interval of 34 ± 9 months and at least 18 months following surgery. The study-specific questionnaire was filled in by all 42 patients and showed that 39/42 (93%) patients with 43/46 (93%) tendons were satisfied (*n* = 37) or very satisfied (*n* = 6) with the treatment. The mean VISA-A score increased significantly from 41.9 ± 18.2 pre-operatively filled in by all patients to 87.7 ± 18.2 post-operatively (*p* < 0.001) filled in by 39 patients.

Twenty-five tendons (20 male and 4 female patients) were operated on with removal of the upper calcaneus, subcutaneous, and retro-calcaneal bursae and the distal part of the plantaris tendon (Figure 1). For twenty tendons, patients were satisfied with the treatment and filled in the two VISA-A scores. Two patients were satisfied, but did not fill in the follow-up VISA-A scores. One patient did not consent to participate in the follow-up and another patient died of an unrelated heart condition, but was satisfied with the treatment at five months post-operatively.

Twelve tendons (nine male and three female patients) were operated on with removal of the subcutaneous bursa and distal part of the plantaris tendon. For eight tendons, patients filled in the two VISA-A scores, and for another two tendons, patients responded to the study-specific questionnaire, but did not complete the post-operative VISA-A. One of these ten patients was not satisfied with the treatment. Two patients did not respond to the follow-up invitation.

Nine tendons (three male and three female patients) were operated on with removal of the subcutaneous bursa alone (Figure 2). Eight of these nine patients were satisfied. For five tendons, patients filled in the two VISA-A scores, and for another three tendons, patients responded to the study-specific questionnaire, but did not complete the post-operative VISA-A. All patients were satisfied with the treatment. One patient did not respond to the follow-up invitation.

Seven tendons (four male and two female patients) were operated on with removal of the subcutaneous bursa and intra-tendinous bone fragments/spurs (Figure 3). For six tendons, patients filled in the two VISA-A scores, and for one tendon, the patient responded to the study-specific questionnaire, but did not complete the post-operative VISA-A. All patients except the one who did not fill in the post-operative VISA-A were satisfied with the treatment.

Two patients had a culture-verified superficial wound infection. These were treated successfully with short-time antibiotics. One patient reported a minor wound rupture that healed successfully without superimposing infection. An additional operation was needed in two patients because of remaining pain from a sharp bone edge located outside the area of the index surgery. Both patients were satisfied at the follow-up and were back to their pre-activity levels.

## 4. Discussion

In this almost 3-year follow-up study of patients operated on for chronic painful insertional Achilles tendinopathy, US- and CD-guided WALANT surgery showed high subjective patient satisfaction rates and good results concerning function in a majority of patients. Patients in the current study mainly represented insurance patients and private patients seeking help for chronic painful insertional Achilles tendinopathy. It was a generally healthy group involved in different sports and recreational activities, and only a few suffered from medical conditions like hypertension and type-2 diabetes. The low mean pre-operative VISA-A scores clearly showed that these patients had major pain and functional disabilities despite various non-surgical treatment regimes.

### 4.1. Ultrasound-Guided Surgery

The US and CD examination is reliable, detects tendon, bursae, and bone pathology [10], and guides the WALANT surgical procedure; the US examination can thus replace the pre-operative use of radiographs and MRIs. The dynamic US examination makes it possible to visualize and examine all tissues in the insertional area and find the exact position of bone spurs and loose bone fragments to guide bedside treatment, including surgery. Patients in the current study had different types of insertional Achilles tendinopathy with various combinations of tissues involved and addressed, but results were generally good at follow-ups regardless of this, and were in accordance with previous reports [19]. There are very few studies on this surgical approach so far. In a recent paper using the same technique, rehabilitation, and follow-up, the study was performed at a county hospital and patients mainly had low activity levels [19], while the current study was performed at a private clinic and patients mainly had high activity levels and sports participation. From the results from these different papers, it appears that both patients with low activity levels and those with high levels of sports activities can have beneficial effects after this type of surgical method.

There is no golden standard surgery for insertional Achilles tendinopathy, and several surgical procedures have been introduced for this condition [12,13,14,15,16,17]. In Sweden, the Nunley procedure using Achilles tendon detachment from the calcaneus and the Keck and Kelly procedure using calcaneal osteotomy are common [13,16,17]. Both methods are performed under general or spinal anaesthesia and include a period of immobilisation followed by a long rehabilitation over several months up to a year. Another method in use is endoscopic removal of the retro-calcaneal bursa and Haglund deformity [5,6,12], but that method does not allow removal of the subcutaneous bursa and intra-tendinous bone formations.

Compared to the Nunley and Keck and Kelly procedures, it seems that the US- and CD-guided WALANT surgery has several potential advantages. US and CD guidance is a reliable diagnostic tool, and the dynamic examination can identify and locate all tissues involved. It further allows for proper positioning of skin incisions and any tenotomy, which can minimise tissue trauma and avoid tendon detachment or iatrogenic accidental tendon injury. The limited tissue trauma and absence of tendon detachment allows for immediate weight-bearing loading and no immobilisation in a plaster cast or orthosis. Furthermore, the operation is performed on otherwise healthy patients under local anaesthesia, thus avoiding any potential risks and side effects of using general or spinal anaesthesia. Finally, the need for sick leave from work varies and depends on the type of occupation, but often there is no need for sick leave at all compared to long sick leave periods required with immobilisation and non-weight-bearing after tendon detachment and osteotomy procedures. However, this is currently unexplored, and further research is needed, especially randomised trials comparing the Nunley and/or Keck and Kelly procedures with this US- and CD-guided WALANT surgery.

### 4.2. Role of the Subcutaneous Bursa?

We found similar and good results in a sub-sample of patients after removal of the subcutaneous bursa alone. Interestingly, a recent study using immunohistochemical analyses of tissue samples in patients suffering from chronic painful insertional Achilles tendinopathy showed that most nerve structures were found in the subcutaneous bursa, followed by the retro-calcaneal bursa and distal Achilles tendon [7]. Therefore, the subcutaneous bursa tissue could alone, or together, be responsible for most of the pain. To the best of our knowledge, there are only a few previous studies reporting on the importance of removing the richly innervated subcutaneous bursa specifically [19,20]. The major negative effect of removal of the subcutaneous bursa is that there is a concomitant loss of skin sensation at the back of the heel. Patients are therefore carefully informed about this before surgery, and during the first six post-operative weeks, patients need to wear a shoe with an open heel. Fortunately, skin sensation becomes increasingly normal over time and no patient has so far reported any complaints about poor skin sensation after completion of the rehabilitation protocol; instead, all patients are relieved that the burning pain located at the back of the heel has resolved.

### 4.3. Complications

We had no major complications after surgery. There were two superficial skin infections (staphylococci in the culture) that were successfully treated with antibiotics, and one minor wound rupture that healed successfully without superimposing infection. As the surgical field is a well-known risk area, we are very careful with wound closure and dressing and use a specific written information sheet for patients regarding how to monitor and manage their wound and wound dressing.

### 4.4. Methodological Considerations

Our study had several possible limitations. First, our study was a prospective and consecutive case series with no control or comparison group. Second, this study reported only mid-term results (minimum follow-up time 18 months, mean follow-up time 34 months); longer-term results, after at least five years, are warranted in the future. Third, there might have been a risk for observer bias, but this was counteracted using an independent foot and ankle surgeon (P.B.) who was not involved in the inclusion or care of patients for follow-ups. Fourth, operating a US probe is user-dependent, and the interpretation of images requires considerable experience with a long learning curve; to minimise bias, all US and CD examinations were standardised and performed by an experienced examiner (H.A.). Fifth, there were six dropouts at follow-up and eight additional cases with missing post-operative VISA-A scores, but seven of these eight patients with missing VISA-A scores were satisfied with the treatment; this should, therefore, have a negligible influence on the overall results. Sixth, a higher number of patients would have been optimal.

## 5. Conclusions

In conclusion, the results from this case series were favourable and showed that the US- and CD-guided WALANT surgical procedure can be a good alternative for treatment of chronic painful insertional Achilles tendinopathy. This novel treatment approach warrants more studies, including randomised trials with comparison against traditional surgical procedures according to Nunley and Keck and Kelly.

## Figures and Tables

**Table 1:** Structured 12-week rehabilitation protocol following US- and CD-guided WALANT surgery for insertional Achilles tendinopathy showing the recommended activity for each week.

**Weeks 1–6:** Mandatory use of a shoe with an open heel to protect the sensitive skin area at the back of the heel. Immediate free range of motion exercise for the ankle and foot. Gradually increased slow-speed walking with full weight bearing allowed immediately, except if there was a tenotomy with intra-tendinous bone removal, when half of bodyweight was recommended in weeks 1–2. Light biking was allowed immediately. **Weeks 7–12:** Introducing adaptation to normal shoes. Gradually increased walking distance and speed. Gradually increased biking distance and speed including heavier intervals. Gradually increased strength training using heel raises and eccentrics. **Weeks 13-forward:** Introduction of light jogging (jogging 50 m, then walking 50 m; jogging 100 m, then walking 50 m; etc.) Introduction of sport-specific exercises other than walking, biking, and strength training.

## Data Availability

Raw data supporting the conclusions of this article will be made available by the authors on request.
